# Best practice: setting up and operating a mid-sized cryo-EM facility

**DOI:** 10.3389/fmolb.2023.1302680

**Published:** 2023-11-27

**Authors:** Xing Meng, Ishara Ratnayake, Martha L. Escobar Galvis, Jason Kotecki, Zack Ramjan, Gongpu Zhao

**Affiliations:** ^1^ David Van Andel Advanced Cryo-Electron Microscopy Suite, Van Andel Institute, Grand Rapids, MI, United States; ^2^ Cryo-EM Core, Core Technologies and Services, Van Andel Institute, Grand Rapids, MI, United States; ^3^ Office of the Cores, Core Technologies and Services, Van Andel Institute, Grand Rapids, MI, United States; ^4^ Information Technology, Van Andel Institute, Grand Rapids, MI, United States

**Keywords:** cryo-EM, core facility, infrastructure, IT-tools, setting up, operation, maintenance

## Abstract

Ever since the resolution revolution in 2013, cryo-electron microscopy (cryo-EM) has become a powerful methodology in structural biology that is especially suited to study the structure of large flexible molecular complexes. Since then, the need of setting up state-of-the-art cryo-EM facilities around the world has increased tremendously. Access to high-end cryo-EM instrumentation is however expensive and requires expertise. The establishment of large cryo-EM centers worldwide, many of which provide academic users free access for both data collection and user training, has been possible with the support of government agencies across the globe. In addition, many universities, and private institutions like the Van Andel Institute (VAI) have made significant investments to establish their own cryo-EM core facilities, ensuring on-site access to their researchers. This paper aims to serve as a blueprint for establishing a new mid-sized cryo-EM facility, as it provides key information based on our experience at VAI and discusses strategies used to optimize routine operation towards high performance and efficiency for single-particle cryo-EM. Information regarding initial planning, selection of equipment as well as the development of IT solutions that were required to improve data collection and analysis are included. In addition, we present an account of the most common issues affecting operation as well as the needs for maintenance over a 6-year period, which can help interested parties to estimate the long-term costs of running this type of facility. Lastly, a brief discussion on the pros and cons of establishing the facility is also included.

## 1 Introduction

From the moment it was first revealed that biological samples could be imaged within vitreous ice (Adrian et al., 1984), it became evident that cryo-electron microscopy (cryo-EM) would play a significant role in the experimental determination of macromolecular structures. The path to its widespread adoption has however, been far from smooth, encountering numerous obstacles along the way. Remarkable milestones, included the development of the single-particle method (Frank et al., 1981; Radermacher et al., 1987; Van Heel, 1987), the first subnanometer-resolution structures of viruses (Conway et al., 1997), and the commercial availability of direct electron detectors (Li et al., 2013; Liao et al., 2013; Amunts et al., 2014). Collectively, these breakthroughs have contributed to the so-called “resolution revolution” that took place in the early 2010s, solidifying the importance of cryo-EM in the field (Kuhlbrandt, 2014; Nogales, 2016).

As cryo-EM has increasingly become a mainstream methodology in structural biology, its wider adoption and accessibility have grown to be critical for every researcher in the field. However, access to high-end cryo-EM instrumentation is not only expensive but also requires specialized personnel. To address these challenges, government agencies across the globe have invested heavily to establish large national cryo-EM centers (Alewijnse et al., 2017; Clare et al., 2017), many of which provide academic users free access for both data collection and user training. In addition, many universities (Walsh et al., 2022) and private institutions like the Van Andel Institute (VAI) have made significant investments to create their own cryo-EM core facilities, ensuring on-site access to instrumentation and expertise that can also be made available at a regional level.

Although our current work has been limited to single particle cryo-EM, our facility provides project-focused support, as we have a close connection with the local research laboratories. Our staff are familiar with the users’ projects being able to follow both the progress and challenges that arise as each project develops. We have created tailored protocols for data collection and user training to meet the specific needs of each user. Our facility also offers access to tools developed in-house, which have been optimized for data collection and analysis. In this article, we outline the establishment of the VAI Cryo-EM Core Facility, including a comprehensive account on how major challenges in its setting up were surmounted. Additionally, we provide an overview of routine operations and the creation of in-house solutions to maximize our efficiency. Our primary objective is to offer the research community a blueprint that can aid in setting up additional regional cryo-EM centers, thereby enhancing access to this powerful technique.

## 2 Materials and methods

### 2.1 Minimum requirements and initial planning

In addition to the initial investment for instrument purchase and installation, the recruitment of staff personnel is of utmost importance. To optimize usage efficiency and minimize instrument downtime our personnel guarantees the implementation of standardized protocols, adequate training, and support, as well as the performance of routine maintenance tasks. Long-term investment is therefore not only necessary for equipment acquisition and maintenance but also to maintain a sufficient staff-to-trainee ratio in the long-term.

Careful consideration of the number and type of electron microscopes required, as well as the allocation of dedicated spaces for each instrument, is essential during the site selection process for a cryo-EM facility. By adopting state-of-the-art technology and optimizing workflows for sample screening and data collection, researchers can maximize the efficiency and resolution capabilities of their cryo-EM endeavors. In contrast to large national cryo-EM centers, in which housing several similar microscopes within a single spacious room optimizes the use of shared infrastructure, in smaller facilities, it is common for each microscope to utilize an individual room. Room size is often limited and there is a need of improved control conditions for 200 and 300 kv microscopes.

### 2.2 Equipment selection

The experimental workflow for cryo-EM encompasses multiple components, including sample preparation, negative stain sample screening, cryo-grid screening, and high-resolution data collection. Based on our experience focusing on single particle cryo-EM, we recommend acquisition of three microscopes (120, 200, and 300 kV). Negative stain sample screening serves as a rapid and accessible method for evaluating sample quality and morphology at room temperature; a 120 kV entry-level microscope is sufficient for this purpose. Also, it is generally recommended to conduct screening and data collection on separate cryo-EM microscopes. Cryo-grid screening can be labor-intensive, and often requires iterative optimization of both the sample and freezing conditions. Thus, this stage can constitute a bottleneck within the cryo-EM workflow. Employing a 200 kV microscope equipped with an autoloader can markedly enhance throughput and it is therefore a good way to overcome this problem. Although, recent advancements in energy filters and camera technology have facilitated the achievement of near-atomic resolutions on 200 kV microscope (Koh et al., 2022), state-of-the-art 300 kV instruments remain the gold standard for collecting the highest resolution data to date (Nakane et al., 2020).

### 2.3 Space considerations and required room settings

Cryo-EM are delicate instruments that need 24/7 support from institutional infrastructure. These instruments are extremely sensitive to environment factors such as temperature, vibration, acoustic noise, humidity, direct (DC) and alternating (AC) electric fields, etc. (David et al., 2006; Sader et al., 2020). In most cases, these instruments are located in the basement without any space below to avoid floor vibration. When microscopes are to be installed on a higher floor, a vibration cancellation system needs to be put in place (Preumont, 2011). It is important to mention that reserving an isolated area of the building for installation of the microscopes in order to avoid excessive traffic is also beneficial. All essential instruments should be connected to an emergency power source. If feasible, it is recommended to separate the power supply and other supporting infrastructure, such as compressed air, from the rest of the building to minimize potential disruptions to services.

**FIGURE 1:**
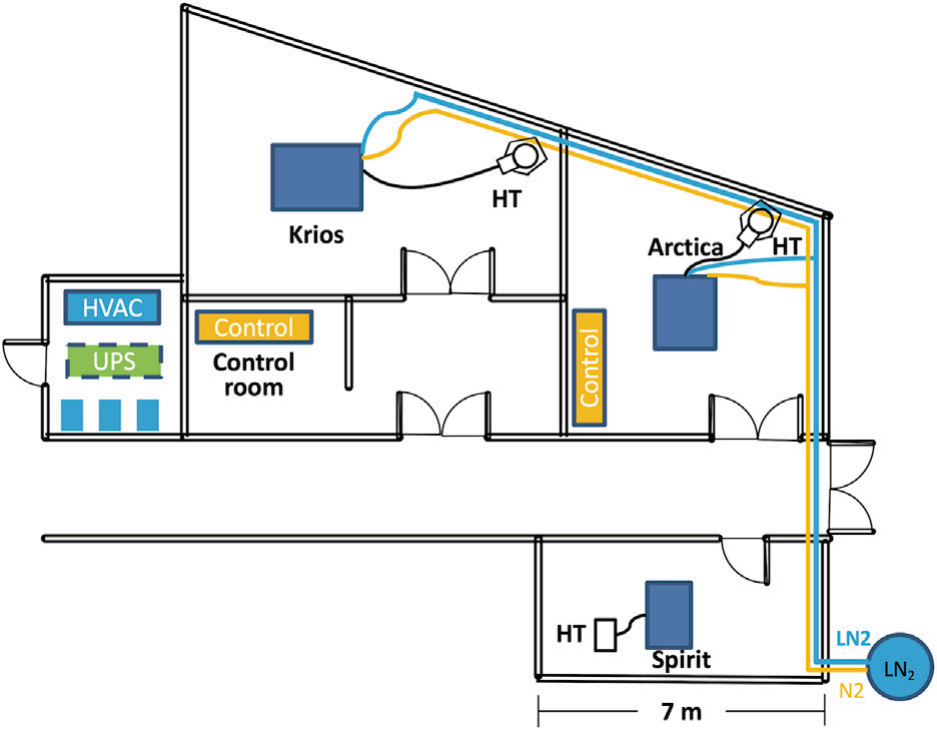
Floor plan of the cryo-EM suite. The three microscopes (Spirit, Arctica and Krios) are housed in three adjacent rooms. The Artica and Krios are connected to a LN_2_ tank located next to the building through vacuum jacketed pipe. The HVAC and three water chillers colored in blue are housed on the first floor of a mechanical shaft. The UPS, colored in green, is housed on the second floor of the same mechanical shaft. The LN_2_ pipe is colored in blue and the N_2_ gas pipe is colored in orange. Scale bar = 7 m. Abbreviations: HVAC, heating, ventilation, and air conditioning; UPS, uninterruptible power supply.

At VAI, the three electron microscopes (EMs) have been placed in separate rooms located next to each other (Figure 1). Six heating, ventilation, and air conditioning (HVAC) units and three water chillers have been placed in a mechanical room next to the EM suite. Furthermore, the EMs need access to pure nitrogen and compressed air, which should be made continuously available. The climate conditions in each microscope room are individually controlled by two water-cooled HVAC units with one running and the other one serving as a backup; the two HVAC units alternate weekly. The room temperature has been set at 21°C with a variation less than 0.3°C (over a 24-h period) and a relative humidity of 40 percent. The HVAC vent has air diffusers distributed around the wall, and the return air vent is located in the center of the ceiling, above the microscope.

To reduce vibration, the Krios is mounted on a 10 ft by 8 ft concrete isolation slab. The Krios room is separated from the corridor by two doors to minimize dust, humidity intrusion and sudden pressure changes. The AC magnetic field is periodically monitored to be 0.2–0.3 mG in horizontal direction, 0.4–0.5 mG in the vertical direction. The DC magnetic field is periodically monitored to be 0.1–0.2 mG in horizontal direction, less than 0.1 mG in the vertical direction.

During operation, both Arctica and Krios microscopes require approximately 200 L of liquid nitrogen (LN_2_) per week. Due to the significant distance from the building’s LN_2_ storage tank, piping LN_2_ to the microscope represented very high costs at the time of installation. Consequently, we decided to use individual 230 L low pressure cryogenic liquid cylinders for each microscope. These cylinders supply LN_2_ and concurrently provide pressurized nitrogen gas. While this configuration has proved reliable over the years, it has had some drawbacks. Occasionally, low-pressure following filling would result in delays in sample loading. Moreover, cylinder filling and transportation requires access to personnel and causes inevitable service interruptions. To overcome these challenges, a dedicated LN_2_ storage tank near the cryo-EM suite with direct LN_2_ connections through vacuum jacketed piping to both the Arctica and Krios microscopes has been constructed, as depicted in Figure 1.

### 2.4 Optimizing IT and network infrastructure

**FIGURE 2:**
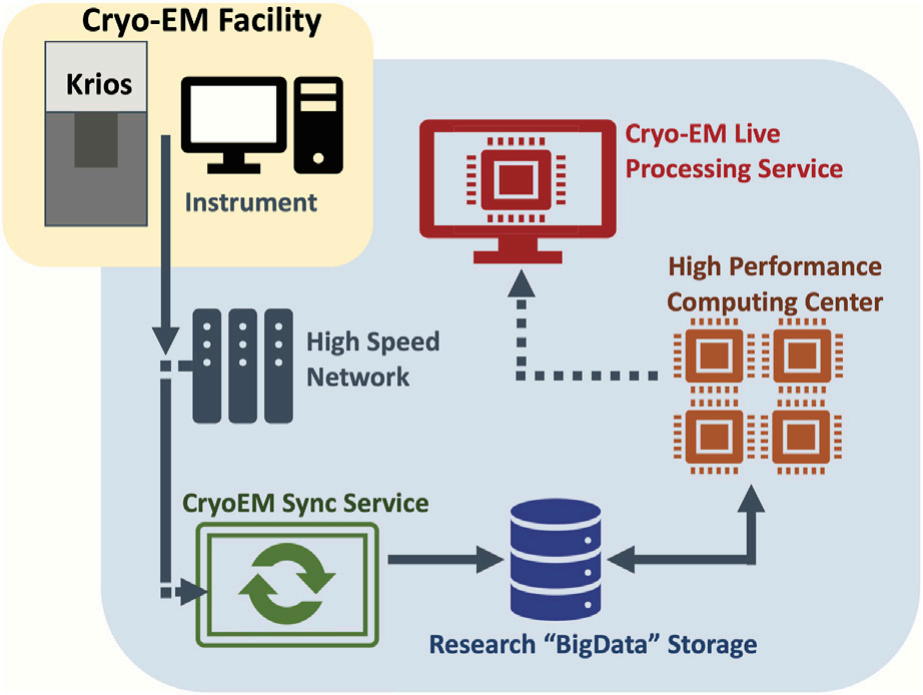
Graphical overview of the data flow from the microscopes through the various phases of storage and processing. The VAI cryo-EM instruments are connected to a central research storage through an automated sync service. Live processing service carries out image processing of the data in real time.

One of the initial IT challenges was accommodating the flow of data from the microscopes through the various phases of storage and processing. Figure 2 provides a graphical overview.

At the beginning we only used the K3 servers supplied by the vendor (Gatan Inc.). Unfortunately, there was little flexibility with this server system. We felt that the local SSD to house data streaming of the instrument was undersized and that the 10 Gbe was not ideal. To work around the limited amount of local storage, we created a Sync Server that runs as a Linux VM and can be accessed as a shared drive. This server transfers data off the K3 servers, as soon as possible, to prevent the filling up of local SSD. One of the issues encountered was poor single-stream performance, i.e., a single file could not be transferred quickly enough. To work around this problem, we have used custom scripts to generate a list of files, and then spawn multiple parallel transfers that happen every 5 min. Also, to prevent overflow files from the K3 server SSD that are older than 4 h are deleted. Our sync server scripts are publicly available on GitHub: https://github.com/VanAndelInstitute/VAICryoTools.

The job of the Sync Server is to move data from the instrument to our “BigData” institutional research storage, in which about 350 TB out of the total 6.5 PB space have been assigned to the cryo-EM facility. Research storage is designed to support demanding workloads with dozens of nodes working in parallel to process a single dataset. This storage system is a Lenovo DSS running IBM SpectrumScale that was deployed in 2021.

Once the data are on Research Storage, they can be accessed by VAI institutional HPC resource, including dedicated GPU nodes. One of these nodes is a specialized Live Processing server that watches for incoming data and analyzes it on the fly. On the node Cryosparc Live is installed. It can process up to ∼500 K3 movies per hour and perform live 2D classification and 3D refinement at the same time. With the stream processing, users could get instant feedback in term of sample quality, ice thickness, protein conformations, etc., which is invaluable when working on challenging samples.

All three microscope-control computers, as well as the camera servers have also been set up to allow remote access. For external access, teamviewer is used by contractors for service checkup, diagnose, remote maintenance. For internal access, the IT department at VAI has deployed a remote access gateway running Apache Guacamole. After logging in, a user can choose whichever instrument they need to monitor and control. To prevent a user from interfering with other’s work at the same time the gateway only allows one user at a time to operate a given instrument. The remote access has enabled access the microscopes during off hours and ensures a smooth 24/7 operation of the cryo-EM facility. This system proved to be especially useful during 2020 and 2021, when access to the labs was restricted due to COVID-19.

### 2.5 Computational aspects required for modeling

The collection and analysis of cryo-EM datasets has been streamlined with recent advances in both hardware and software. Still, cryo-EM facilities generate substantial volumes of data; we normally generate data in the range of tens terabytes per day. The ingest and storage of these data demands a high-performance file system capable of making data accessible to many compute nodes performing analysis in a high-performance computing environment. At VAI, we have been using IBM SpectrumScale, a parallel filesystem, to support the computation storage needs of cryo-EM users. The SpectrumScale filesystem is integrated with our HPC computational environment such that compute nodes can read data at up to 4 GB per second. Overall, VAI’s HPC is a heterogeneous environment including various types of resources (e.g., dedicated high memory nodes, GPU nodes or high-core-count nodes) that uses Slurm job scheduler to allocate resources.

After the data are generated and transferred into the central storage, each user creates their own image processing pipeline. At VAI, image processing and cryo-EM density map reconstruction, are mainly done through Relion (Zivanov et al., 2022) or Cryosparc (Punjani et al., 2017). In terms of usage GPU nodes are the most used resource (currently with 0.5 TB of memory and multiple Nvidia RTX A6000 cards) for cryo-EM analysis. For large-scale modelling using molecular dynamics, high-core count, CPU-only nodes in parallel, using MPI (message passing interface), are preferred.

## 3 Operation

### 3.1 Setup of equipment and routine operation

Our facility has a dedicated core team that has been entrusted with the meticulous alignment of the microscope, the initialization and calibration of data collection software, and the assurance of peak microscope performance. To uphold our rigorous quality control standards, each session is prefaced with a thorough performance check of the microscope using thon rings from quantifoil or C-flat holy carbon grids. To visualize the Thon rings, the electron beam is directed onto the carbon area for 1–2 min, effectively removing vitreous ice. Astigmatism correction and coma-free alignment are meticulously executed in SerialEM or comparable data collection software. A super-resolution movie is then recorded, typically with a total electron dose of 40–60 e-/Å^2^ at a defocus of −0.7 µm, followed by drift correction. The resultant power spectrum, derived from the sum of the movie frames showcasing Thon rings, serves as a rapid assessment of both the microscope’s performance, and grid quality. Common issues identified with this approach include anisotropic resolution limitations, due to specimen drift or vibration (Kuo, 2007), or isotropic attenuation of the Thon rings at higher spatial frequencies, because of carbon thickness (Tichelaar et al., 2020). We aim to observe Thon rings extending to at least 2.5 Å prior to initiating data collection.

Furthermore, to confirm the microscope’s resolution capabilities as well as the pixel size calibration, we regularly collect high-resolution data using β-galactosidase (Bartesaghi et al., 2015) as standard samples for single particle reconstruction. Other standard samples commonly used are 20S proteasome (Alewijnse et al., 2017), apoferritin (Alewijnse et al., 2017; Sader et al., 2020), or tobacco mosaic virus (Kandiah et al., 2019). All of them have been routinely employed across cryo-EM labs or facilities to evaluate the entire single particle reconstruction workflow–from sample preparation and data collection to image processing–for optimization purposes. Notwithstanding, a well-equipped facility is expected to consistently achieve high-resolution reconstructions with these standard specimens. At our facility, we achieve routine reconstructions at 2.0–2.3 Å resolution on the Krios for β-galactosidase. We normally collect super resolution movies on K3 with a nominal magnification of 105,000 (by SerialEM), using the Bioquantum energy filter set to zero-energy-loss mode and an energy slit width of 20 eV. Our total accumulative electron dose is ∼60 e^−^/Å^2^ fractioned over 50 subframes. Prior to data collection, we measure ice thickness using energy filter as in Rice et al. (2018) and only select grid areas with a of thickness ≤40 nm for data collection.

Being a mid-sized facility with limited staff capacity, our foremost goal is to instill in every user the proficiency required to independently operate the microscope and initiate data collection. Most users can navigate the cryo-EM workflow autonomously, with core staff on standby for occasional support. It is noteworthy that at VAI, cryo-grid screening is exclusively conduct on the Arctica system.

We have opted for a flexible approach that does not restrict access to Arctica-time to individual labs. All users are offered the opportunity to book either 6-h morning or 18-h afternoon/overnight sessions. Morning sessions serve predominantly for quick freezing condition screening and acquisition of smaller datasets of several hundred images. Conversely, overnight sessions are earmarked for the collection of smaller datasets across multiple grids or larger datasets of 2,000 to 3,000 images from a single grid, facilitating analysis of 2D class averages and attainment of medium-resolution reconstruction.

Only upon optimization of a sample has been achieved, users can submit an online request for access to Krios time. This time is typically allocated in 48-h slots but can be extended to up to 4 days for if samples present greater challenges. With the efficiency of SerialEM, the Krios system is capable of amassing roughly 400 images per hour, culminating in an impressive collection of approximately 18,000 images after a 2-day session.

### 3.2 User training

The VAI Cryo-EM Facility, initially established to serve internal laboratories, has since expanded its services to accommodate external users. While most of our users are self-sufficient, our staff is always available to offer assistance and training when necessary. We have developed a comprehensive, multi-step training program tailored to meet the needs of individuals user.

Our robust training program empowers most users to navigate the cryo-EM workflow autonomously, with core staff on standby for occasional support. New users, with no prior experience, undergo an initial training phase that covers negative stain sample preparation, electron microscopy principles, microscope operation, and safety procedures. Following this training, each novice user must complete 40 h of hands-on experience using the Spirit microscope and pass a proficiency assessment to become certified for independent operation. Experienced users may bypass the 40-h requirement and proceed directly to the certification test.

Once certified on the Spirit microscope, users are eligible to request training on the Arctica system. This advanced training encompasses sample vitrification, grid loading, microscope operation, direct detection camera operation, and automated data collection utilizing software such as EPU and SerialEM, both integral to our cryo-EM workflow.

Because the Krios microscope is completely dedicated to high-resolution data collection, we think that granting unrestricted access would be counterproductive. Therefore, the core facility does not provide training on the Krios microscope. Currently, core staff members are responsible for alignment, tuning the energy filter, and conducting quality checks. Users are permitted to set up data collection runs but are not granted extensive access to the Krios microscope.

### 3.3 Maintenance

In a proactive way, good maintenance can minimize the equipment down time, reduce running cost and it is a key to sustain the work efficiency. Tracking various information can really help to fulfil this task.

#### 3.3.1 Integrated tracking system

Cryo-EM facilities, supported by institutional infrastructure, are susceptible to disruptions of internal and external origin. Factors such as planned or unplanned power outages as well as considerable fluctuations in building cooling water temperature can impact the microscope chiller, which often results in downtime. These external events, in conjunction with internal component failures, can easily instigate a cascade of events leading to significant increases in downtime. Thus, to maintain a cutting-edge cryo-EM facility, it is imperative to implement a thorough and methodical approach. A vital element for us, involved the establishment of an integrated tracking system to monitor key parameters, including chill water temperature, pH value, pressure, and vacuum levels. The system, utilizing Zoho forms, captures the facility’s status by integrating manual input and photographic evidence (Table 1). By consistently monitoring these parameters, we have proactively identified and addressed potential issues, including those arising from external events (i.e., power outages, building cooling water temperature shifts) over the last 6 years. Thanks to this approach, we have been able to ensure an 87 and 89 percent uptime for Krios and Arctica, respectively.

**TABLE 1:** Types of operational information collected over the last 6 years. Database powered by Zoho forms.

Forms	Update	Parameters
Krios/K3/GIF status	Bi-monthly	Temperature, Vac, settings, water flow rate
Arctica/K2 status	Bi-monthly	Temperature, Vac, settings, water flow rate
Spirit status	Bi-monthly	Temperature, Vac, settings, water flow rate
Sample prepare room	Bi-monthly	Gases (N_2_, O_2_ and Ethane) levels
Chillers	Bi-monthly	Water level, temperature, and pH value
Equipment Accident Report	Per event	Instrument, date, issue, service process and outcome
Spare parts	Per item	Photos and locations
Spare tools, gases, backup tools	Per item	Photos and locations

#### 3.3.2 Downtime analysis and extended trend evaluation

Investigating downtime incidents is critical for the maintenance of a cryo-EM facility. Classifying these incidents based on root causes, like internal component failures and external disruptions, can assist in anticipating and averting future equipment failures, and ultimately help to reduce interruptions to ongoing research activities.

**FIGURE 3:**
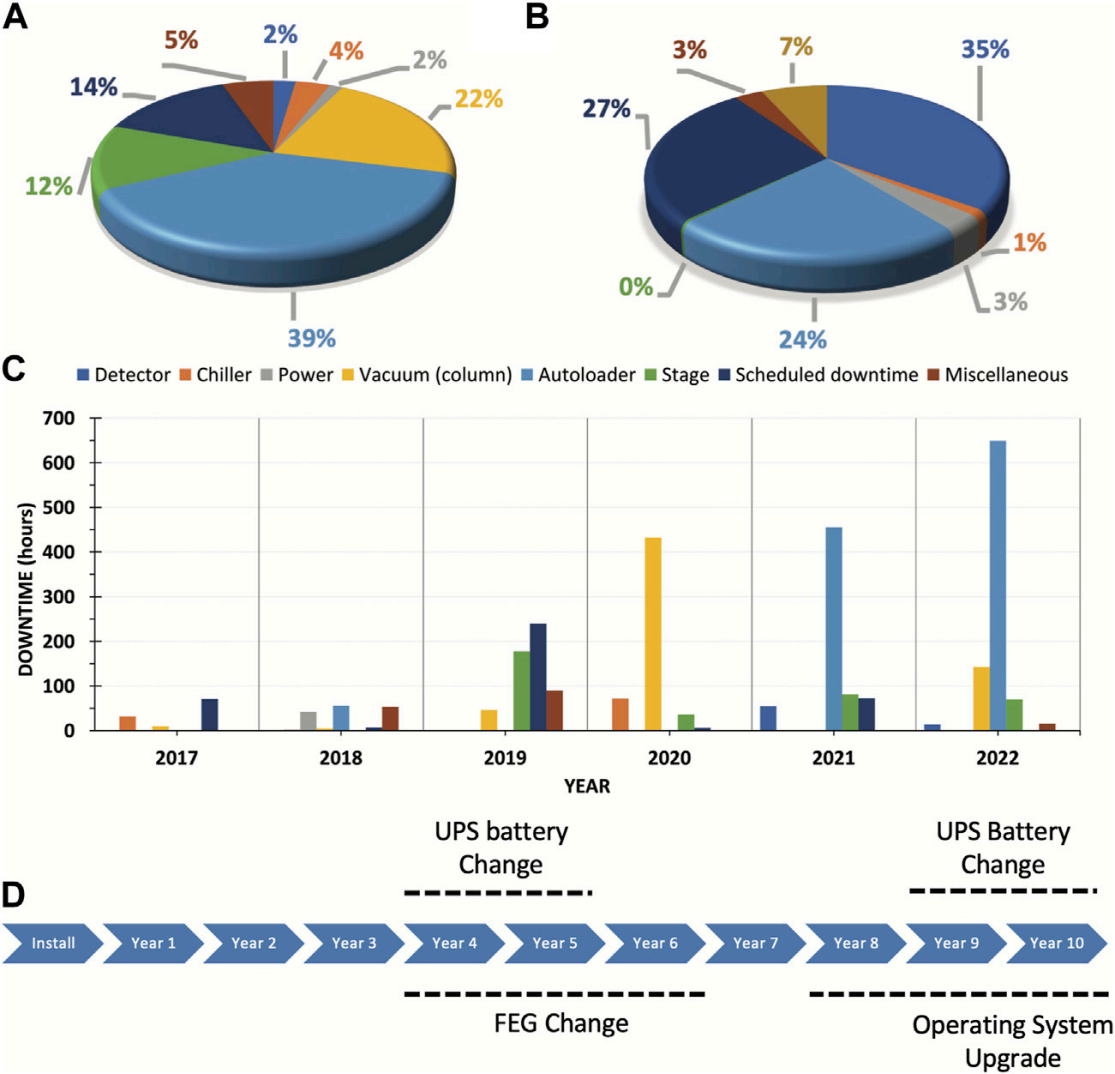
Documented events throughout 6 years of Arctica and Krios usage. A&B. Downtime distribution according to type of event for Arctica **(A)** and Krios **(B)**. **(C)** Arctica downtime events per year from 2017 to 2022. **(D)** Actual and projected significant maintenance milestones over a 10-year period. Abbreviations: FEG, field emission gun; UPS, uninterruptible power supply.

Thus, throughout 6 years of Arctica and Krios usage, we have documented events (organized into distinct categories based on the downtime associated with each event and summarized in Figures 3A, B). The top three categories involved the autoloader, vacuum, and stage. While the vacuum issue may be specific to the individual microscope, autoloader and stage issues are quite prevalent.

##### 3.3.2.1 Autoloader patterns and challenges

Extended trend analysis over the past 6-year period has exposed recurring issues requiring further scrutiny. Autoloader problems typically arise after 3–4 years of use, as this intricate machinery is susceptible to malfunction and user errors. The three primary subcategories of downtime incidents consisted of mechanical problems, electrical board issues, and vacuum leaks. The most common issue was failed initialization of the autoloader, which in most of the cases was due to failure in the control board. In other cases, the failed initialization was due to something unrelated such as a broken stage wire. On two occasions, the cassette failed to dock because the cassette griper arm was compromised (once the arm was loose and another time the arm was slightly bended). We also documented two rare events in which failed initialization was the result of the cartridge griper not being able to retrieve the grids due to a damaged tip. Extended downtime during years 5 and 6 (Figure 3C) resulted from damage to either the cartridge griper or cassette griper. These are usually challenging parts to replace since they can only be directly shipped from the factory in Europe. Unfortunately, in our case such replacement was aggravated by supply chain delays associated with the pandemic. The most common user errors we have come across involved improperly loaded grids into the cassettes. Such events can eventually affect either the autoloader or the column, potentially resulting in other issues like vacuum leaks at the docking valve or obstructed views if within the column.

##### 3.3.2.2 Stage problems and resolution

Our analysis demonstrates that stage issues have consistently occurred from the third-year after installation. We observed that the stage displays jittering behavior before it eventually stops functioning. Given that the Arctica is extensively used for screening and operates 24/7, it is important for us to be able to anticipate this type of mechanical problems. The predominant issues observed entailed malfunctioning stage control boards, encoders, or motors governing the stage motion. We recommend maintaining a clean environment, to prevent dust accumulation, and adequate lubrication of all moving parts. Please note that the open enclosure design of the Arctica leads to noticeable dust accumulation on the floor beneath it (a challenge not encountered with the Krios). In our case, the heightened dust accumulation is likely due to heavier traffic within the Arctica room, as we placed the control station in the same room due to space constraints. Ideally, the control station should be placed in a separate room. Additionally, annual preventative maintenance including an inspection of the stage could be helpful to detect potential problems.

#### 3.3.3 Proactive preparation for major maintenance events

Moreover, foreseeing and preparing for significant maintenance events within the first 10 years of operation is crucial (Figure 3D). These events may encompass field emission gun (FEG) replacements, uninterruptible power supply (UPS) battery changes, and essential software upgrades. Proactively scheduling these events facilitates maintaining peak performance and minimizes downtime.

## 4 Conclusion

### 4.1 Performance

The success of the VAI Cryo-EM Core Facility supporting the research of several labs using single-particle cryo-EM has been clearly documented over the past few years. We have supported the work of labs interested in uncovering the molecular machinery behind eukaryotic DNA replication (Yuan et al., 2020a; Yuan et al., 2020b), investigating the bacterial proteasome system in *Mycobacterium tuberculosis* (Xiao et al., 2022), and determining the structure of membrane-embedded enzyme complexes, such as the oligosaccharyltransferase complex (Bai and Li, 2019).

Over the years we have met the needs of our users, creating customized workflows, tailored to their specific projects. We also provide quick access to the equipment and the possibility of training for graduate students, postdoctoral fellows, and technicians. Quick access to state-of-the-art facilities is essential for the successful completion of highly competitive, time-sensitive projects. For example, the determination of the structure of the human lipid-gated cation channel TRPC3 (Fan et al., 2018), a key protein in store-operated calcium entry and calcium homeostasis. This project was completed in 6 months from project launch to the publication of the manuscript. Also, when work was being carried out to determine the structure of ATP-bound AMPK, the crystal structure resolution could not be improved. After a series of measures to stabilize the complex that allowed the use of cryo-EM, researchers at VAI were able to determine two structures (at 3.48 and 3.92 Å), enabling the unambiguous assignment of the kinase domain, core domain, and amino acid 180-203 of the β-linker (Yan et al., 2021).

### 4.2 Limitations

Limitations include additional costs for users as there are internal fees for usage at our core facilities. Such costs are not unusual but considering that usage in the national centers is free of cost, they represent an economic burden for the labs at this moment. It is noteworthy that our core facility extends pilot study opportunities at no cost, aiding labs in gathering preliminary data for grant applications. Also, since the capacity of our facility is restricted by the number of instruments and the fact that internal users must be prioritized, the possibility of offering services to external users has been limited. Furthermore, our ability to regularly upgrade instruments to pursue better resolution as well as improved sample preparation (e.g., more stable energy filters, next-generation electron detectors or a novel sample vitrification robot), is dependent of additional funding. The same applies to implementing new technologies that have not gained enough interest from our users. Unfortunately, if we lack a minimal number of users, the costs associated with acquiring a new instrument and establishing a new technology are simply not justifiable. We believe that over time, this can lead to an expanding disparity in technological capabilities when compared to national centers.

### 4.3 Concluding remarks

Cryo-EM has emerged as a powerful instrument in the realm of structural biology and drug discovery. The necessity for access to advanced cryo-EM facilities is paramount for numerous laboratories. Our aim was therefore to provide a blueprint for the creation and operation of a middle-sized cryo-EM facility that has proven efficient for single particle cryo-EM over the last 6 years.

The establishment of such cryo-EM facility required strategic planning and a long-term commitment, as the large costs associated with their establishment warrant especial consideration. The acquisition of appropriate instruments and the construction or modification of rooms to meet the stringent environmental requirements for cryo-EM are fundamental prerequisites for a successful facility. These steps can help circumvent potential issues in the future. A high-throughput cryo-EM facility is also dependent of a well-structured IT infrastructure. At VAI, we have developed an IT framework that should be beneficial to many new regional facilities with a similar scope. Additionally, we have instituted an efficient workflow to monitor the status of the microscopes and that has minimized downtime since their installation.

We strongly believe that regional cryo-EM facilities can act as hubs for dynamic structural biology communities and can be key in the training the next-generation of microscopists, thereby propelling the evolution and growth of the field.

## Data Availability

The original contributions presented in the study are included in the article/Supplementary Material, further inquiries can be directed to the corresponding authors.
